# CAXII Is a Sero-Diagnostic Marker for Lung Cancer

**DOI:** 10.1371/journal.pone.0033952

**Published:** 2012-03-16

**Authors:** Makoto Kobayashi, Toshihide Matsumoto, Shinichiro Ryuge, Kengo Yanagita, Ryo Nagashio, Yoshitaka Kawakami, Naoki Goshima, Shi-Xu Jiang, Makoto Saegusa, Akira Iyoda, Yukitoshi Satoh, Noriyuki Masuda, Yuichi Sato

**Affiliations:** 1 Department of Applied Tumor Pathology, Graduate School of Medical Sciences, Kitasato University, Kanagawa, Japan; 2 Department of Molecular Diagnostics, School of Allied Health Sciences, Kitasato University, Kanagawa, Japan; 3 Biomedicinal Information Research Center, National Institute of Advanced Industrial Science and Technology, Tokyo, Japan; 4 Department of Pathology, School of Medicine, Kitasato University, Kanagawa, Japan; 5 Department of Thoracic and Cardiovascular Surgery, School of Medicine, Kitasato University, Kanagawa, Japan; 6 Department of Respiratory Medicine, School of Medicine, Kitasato University, Kanagawa, Japan; Dana-Farber Cancer Institute, United States of America

## Abstract

To develop sero-diagnostic markers for lung cancer, we generated monoclonal antibodies using pulmonary adenocarcinoma (AD)-derived A549 cells as antigens by employing the random immunization method. Hybridoma supernatants were immunohistochemically screened for antibodies with AMeX-fixed and paraffin-embedded A549 cell preparations. Positive clones were monocloned twice through limiting dilutions. From the obtained monoclonal antibodies, we selected an antibody designated as KU-Lu-5 which showed intense membrane staining of A549 cells. Based on immunoprecipitation and MADLI TOF/TOF-MS analysis, this antibody was recognized as carbonic anhydrase XII (CAXII). To evaluate the utility of this antibody as a sero-diagnostic marker for lung cancer, we performed dot blot analysis with a training set consisting of sera from 70 lung cancer patients and 30 healthy controls. The CAXII expression levels were significantly higher in lung cancer patients than in healthy controls in the training set (P<0.0001), and the area under the curve of ROC was 0.794, with 70.0% specificity and 82.9% sensitivity. In lung cancers, expression levels of CAXII were significantly higher in patients with squamous cell carcinoma (SCC) than with AD (P = 0.035). Furthermore, CAXII was significantly higher in well- and moderately differentiated SCCs than in poorly differentiated ones (P = 0.027). To further confirm the utility of serum CAXII levels as a sero-diagnostic marker, an additional set consisting of sera from 26 lung cancer patients and 30 healthy controls was also investigated by dot blot analysis as a validation study. Serum CAXII levels were also significantly higher in lung cancer patients than in healthy controls in the validation set (P = 0.030). Thus, the serum CAXII levels should be applicable markers discriminating lung cancer patients from healthy controls. To our knowledge, this is the first report providing evidence that CAXII may be a novel sero-diagnostic marker for lung cancer.

## Introduction

Lung cancer is the leading cause of cancer death, comprising 13% (1.6 million) of the total cancer cases and 18% (1.4 million) of the cancer deaths in the world in 2008 [1], [2].

Tumor markers have been detected in sera, urine, and tissues from patients with malignant tumors, and can be used for an exact diagnosis, discrimination of benign or malignant tumors, follow-up after therapies, and prediction of the patient's outcome. At present, some sero-diagnostic markers are used for lung cancer, such as carcinoembryonic antigen (CEA) and sialyl Lewis X antigen (SLX) for adenocarcinoma (AD), and cytokeratin 19 fragment (CYFRA) and squamous cell carcinoma antigen (SCCa) for squamous cell carcinoma (SCC) [3]. The positive rates of CEA, SLX, CYFRA, and SCCa are reportedly 57, 40∼50, 50∼60, and 60∼80%, respectively. However, it has been reported that these markers do not show sufficient tumor or organ specificities; for example, SLX can show false-positive results in the presence of pulmonary tuberculosis and pulmonary fibrosis, and CYFRA can elevate with interstitial pneumonia and renal failure.

Antibodies are usually developed using purified proteins or synthetic peptides. We have exhaustively generated monoclonal antibodies (MoAbs) against various tumor-associated proteins using the pulmonary AD-derived A549 cell as an antigen with the random immunization method [4], and over 1,000 MoAbs have been obtained [5]. This method is expected to generate antibodies against proteins with tumor-specific post-translational modifications, which are difficult to obtain by conventional immunization methods.

Carbonic anhydrase XII is a transmembrane zinc metalloenzyme that catalyzes the reversible hydration of carbon dioxide to form bicarbonate (H_2_O+CO_2_⇔H^+^+HCO_3_^−^), and is a member of the alpha carbonic anhydrase (CA) family. CAXII has been proposed to be involved in the acidification of the extracellular microenvironment, which is suitable for rapid tumor growth. CAXII overexpression was initially detected in renal cell carcinoma, and subsequent studies confirmed its expression in various human cancers, such as diffuse astrocytoma, breast, pancreatic, and ovarian carcinoma, as well as in non-small cell lung cancer (NSCLC) [6]–[11]. Its expression was influenced both by factors related to differentiation and hypoxia in breast cancer *in vivo*, and was associated with a more favorable prognosis in invasive breast carcinoma patients [12]. Higher CAXII expression was also correlated with a better overall and disease-specific survival in patients with resectable NSCLC [13]. However, no study has clarified CAXII in sera and its clinical utility as a sero-diagnostic marker for patients with malignant tumors.

In this study, the specificity of the obtained anti-CAXII antibody was confirmed by immunohistochemistry (IHC) and immunoblotting with lung cancer cell lines and lung cancer tissues. To further confirm its utility as a sero-diagnostic marker, CAXII levels in sera from patients with lung cancer were studied by dot blot analysis.

## Materials and Methods

### 1. Cell lines

The A549 and LC-2/ad cells derived from lung AD were purchased from the Japanese Cancer Research Resources Bank (Tokyo, Japan) and RIKEN BioResource Center (Ibaraki, Japan), respectively. The RERF-LC-AI cells derived from lung SCC was purchased from the RIKEN BioResource Center. The N231 cells derived from SCLC were purchased from the American Type Culture Collection (Rockville, MD, USA). LCN1, a large cell neuroendocrine carcinoma (LCNEC) line, was established in our laboratory [14]. These cells were grown in RPMI-1640 medium (SIGMA, Steinheim, Germany) supplemented with 10% fetal bovine serum (FBS; Biowest, Miami, FL, USA), 100 units/ml of penicillin, and 100 µg/ml of streptomycin (GIBCO, Auckland, New Zealand). After harvesting and washing twice with phosphate-buffered saline without divalent ions (PBS-), sub-confluent cells were stored at −80°C for proteomics analysis or fixed in 10% formalin and embedded in paraffin for immunohistochemistry. A549 cells were also AMeX-fixed [15] for immunohistochemical screening. The SP2/O-Ag14 cells derived from a mouse myeloma were purchased from the RIKEN BioResource Center, and were grown in RPMI-1640 medium supplemented with 1× 8-azaguanine (50× Hybri-Max, SIGMA), 10% FBS, penicillin, and streptomycin.

### 2. Ethics statement

All samples were collected in accordance with the ethical guidelines and written consent mandated, and this study was approved by the Ethics Committee of Kitasato University School of Medicine. All patients and healthy controls were approached based on approved ethical guidelines, and those who agreed to participate in this study were required to sign consent forms. Patients could refuse entry and discontinue participation at any time. All participants provided written consent.

#### 2.1. Sera

Sera from 70 patients with lung cancer (AD: 29, SCC: 21, SCLC: 17, and LCNEC: 3) and 30 healthy controls were used in the training set. In addition, a validation set consisting of sera from 26 patients with lung cancer (AD: 20, SCLC: 5, and LCNEC: 1) and 30 healthy controls was also studied. The clinicopathological characteristics of the patients data are summarized in Table 1.

**Table 1:** Clinicopathological characteristics of the patients.

Characteristics		Training set (N = 70)	Validation set (N = 26)
Age	<70	40 (57.1%)	19 (73.1%)
	≧70	30 (42.9%)	7 (26.9%)
Gender	Male	52 (74.3%)	16 (61.5%)
	Female	18 (25.7%)	10 (38.5%)
Stage	I	19 (27.2%)	17 (65.4%)
	II	11 (15.7%)	2 (7.7%)
	III	26 (37.1%)	4 (15.4%)
	IV	14 (20.0%)	3 (11.5%)
Tumor differentiation	Well	7 (13.2%)	11 (52.4%)
(NSCLC)	Moderate	10 (18.9%)	5 (23.8%)
	Poor	18 (34.0%)	4 (19.0%)
	Unknown	18 (34.0%)	1 (4.8%)
Tumor size	<3 cm	24 (34.3%)	15 (57.7%)
	≧3 cm	45 (64.3%)	6 (23.1)
	Unknown	1 (1.4%)	5 (19.2)
Nodal status	N0	23 (32.9%)	18 (69.3%)
	N1	12 (17.1%)	1 (3.8%)
	N2	23 (32.9%)	5 (19.2%)
	N3	12 (17.1%)	2 (7.7%)
Distant metastasis	M0	56 (80.0%)	23 (88.5%)
	M1	14 (20.0%)	3 (11.5%)
Histological type	AD^a^	29 (41.4%)	20 (77.0%)
	SCC^b^	21 (30.0%)	0 (0.0%)
	SCLC^c^	17 (24.3%)	5 (19.2%)
	LCNEC^d^	3 (4.3%)	1 (3.8%)

a Adenocarcinoma.

b Squamous cell carcinoma.

c Small cell lung carcinoma.

d Large cell neuroendocrine carcinoma.

Patient sera were collected at Kitasato University Hospital, and healthy control sera were provided by Kyowa Medex Co., Ltd. (Tokyo, Japan) and kept at −80°C until use.

### 3. Generation of monoclonal antibodies

A549 cell lysate was prepared with PBS(-) using an ultra-sonic homogenizer (UH-50; SMT Company, Tokyo, Japan). Five-week-old female BALB/c mice were immunized intra-peritoneally with 50 mg wet- weight of A549 cell lysate in 500 µl of PBS(-) 3 times with a two-week interval. The antibody titer was tested by IHC using 100-times diluted sera from the immunized mice as the first antibody on AMeX-fixed A549 cells. Three days prior to cell fusion, the animal with the highest titer was intra-peritoneally boosted by the same amount of A549 lysate. Hybridoma preparation and IHC screening with AMeX-fixed A549 cells were previously described [4], [5].

### 4. Proteomics analysis

#### 4.1. Sodium dodecyl sulfate-polyacrylamide gel electrophoresis (SDS-PAGE)

Proteins were extracted from each of A549, LC-2/ad, RERF-LC-AI, N231, and LCN1 cells with detergent lysis buffer [16] using an ultra-sonic homogenizer. Ten µg each of extracted proteins were boiled and separated by SDS-PAGE with 10% polyacrylamide gel at a constant current of 20 mA. After SDS-PAGE, proteins in gels were transferred to a polyvinylidene difluoride (PVDF) membrane (Millipore Corp., Billerica, MA, USA) for immunoblotting.

#### 4.2. Immunoblotting

Blotting membranes were blocked with 0.5% casein from bovine milk (Sigma, St. Louis, MO, USA) for 30 min at RT. The membranes were then reacted with non-diluted hybridoma supernatant for 1 hr at RT, followed by incubation with 1,000-times diluted horseradish peroxidase-conjugated rabbit anti-mouse IgG polyclonal antibody (Dako, Glostrup, Denmark) with 0.025% Casein for 45 min at RT. Finally, signals were developed using Immobilon Western HRP reagent (Millipore Corp.).

#### 4.3. Determination of antibody isotype

To determine the isotype of the established KU-Lu-5 antibody, we used the IsoStrip™ Mouse Monoclonal Antibody Isotyping Kit (Roche Diagnostics, Mannheim, Germany) according to the manufacturer's instructions.

#### 4.4. Immunoprecipitation

The immunoprecipitation method used in this study was previously described [17]. In brief, A549 cells were washed with PBS (-) and treated with radioimmunoprecipitation assay (RIPA) buffer containing Complete-mini EDTA-free (Roche Diagnostics) on ice for 30 min. After centrifugation at 15,000 rpm for 30 min at 4°C, the supernatant was collected and precleared with protein G sepharose (50% slurry) (GE Healthcare Bio-Sciences Corp., Piscataway, NJ, USA) at 4°C overnight. To conjugate the primary antibody, 250 µL of primary antibody (KU-Lu-5 hybridoma supernatant) and 20 µL of protein G sepharose beads suspended in RIPA buffer were incubated with mixing at 4°C overnight. After centrifugation, the antibody-sepharose conjugate and 500 µg of total cellular protein from the precleared supernatant were incubated with mixing at 4°C for 4 hrs. The immunoprecipitates were collected by centrifugation at 15,000 rpm for 5 min at 4°C. After washing four times with RIPA buffer, the supernatant was carefully removed and the pellets were resuspended in 15 µL of 1×Laemmili's buffer. Then, 15 µL of samples were boiled and separated by SDS-PAGE with 10% polyacrylamide gel. After SDS-PAGE, gels were Zn-stained with the Negative Gel Stain MS kit (Wako Pure Chemical, Tokyo, Japan) according to the manufacturer's instructions.

#### 4.5. Identification of antigen protein

*4.5.1. In-gel digestion.* The protein spot was excised from the SDS-PAGE gel and minced to 1 mm^3^, destained with destaining solution (Wako Pure Chemical), dehydrated with 100% (v/v) ACN, and dried under vacuum conditions. Tryptic digestion was performed with a minimal volume of digestion solution which contained 20 ng/µl of trypsin (Trypsin Gold, Mass Spectrometry Grade, Promega, Madison, WI, USA) and 25 mM NH_4_HCO_3_ for 24 hrs at 37°C. After incubation, digested protein fragments eluted in solution were collected, and gels were washed once in 5% (v/v) trifuloroacetic acid /50% (v/v) ACN and collected in the same tube.

*4.5.2. Protein identification.* The collected peptide fragments were analyzed using autoflex III matrix-associated laser desorption/ionization-time of flight/time of flight mass spectrometry (MALDI-TOF/TOF MS; Bruker Daltonik, Bremen, Germany). A disposable plate, spotted α-cyano-4-hydroxycinnamic acid matrix for samples, and PAC Peptide Calibstandard for calibration (Prespotted AnchorChip 96 set for Proteomics, Bruker Daltonik) were used. Peptide mass fingerprints (PMF) were measured, and then a few peaks obtained from PMF were further measured for their tandem mass spectra as parent masses. MASCOT (http://www.matrixscience.com) using the IPI Human database (93,289 sequences; 36,994,704 residues), released on 3 May, 2011 (http://www.matrixscience.com), was used to determine proteins from PMF and tandem mass data.

### 5. Immunoblot analysis with recombinant CAXII protein

Recombinant CAXII protein and Venus protein as a negative control with GST-tag were prepared using a wheat germ cell-free system [18]. Fourteen µg each of recombinant CAXII and Venus proteins were boiled and separated with SDS-PAGE, followed by immunoblotting with KU-Lu-5 antibody, as mentioned in 2.4.1.

### 6. Immunohistochemical staining

Three-µm-thick sections, made from 10% formalin-fixed and paraffin-embedded lung cancer cell lines and 37 surgically resected lung cancers (AD: 28, SCC: 9) were deparaffinized in xylene, rehydrated in a descending ethanol series, and then treated with 3% hydrogen peroxide for 20 min. After the antigen was retrieved by autoclaving in 0.01 mol/L citrate buffer (pH 6.0) with 0.1% Tween 20 at 121°C for 10 min, the sections were reacted with non-diluted KU-Lu-5 hybridoma supernatant for 16–18 hrs at room temperature (RT). After rinsing in TBS three times for 5 min each, the sections were reacted with ChemMate Envision reagent (Dako) for 30 min at RT. Finally, the sections were visualized with Stable DAB solution (Invitrogen Corp.) and counterstained with Mayer's hematoxylin.

### 7. Dot blot analysis

#### 7.1. Sample preparation

*7.1.1. Removal of albumin and IgG from serum samples.* The removal of albumin and IgG from sera was performed using a ProteoExtract Albumin/IgG Removal kit (Merck, Darmstadt, Germany) according to the manufacturer's instructions. A 60-µL sample of each sera was diluted with 540 µL of binding buffer, and allowed to pass the column by gravity flow. The flow-through fraction was collected in a collection tube. To wash the column, binding buffer was allowed to pass the column by gravity flow. The flow-through fraction was collected in the same collection tube.

*7.1.2. Desalting and concentration by ultrafiltration.* The albumin- and IgG-depleted samples were buffer-exchanged and concentrated using 10-kDa molecular-weight cut-off ultra-filtration VIVASPIN 2 (Sartorius, Gottingen, Germany). The samples were centrifuged at 6,000×g at 4°C until less than 100 µL, and then the buffer was exchanged for PBS (-) with concentration at 6,000×g at 4°C until concentrated to less than 50 µL. The concentrated samples were adjusted to a final volume of 60 µL with PBS (-).

#### 7.2. Dot blot analysis

One µl each of albumin- and IgG-depleted samples diluted to 1∶20 with PBS(-) and mouse IgG (purified in our laboratory) for a positive control were spotted on a PVDF membrane (Millipore Corp.) using the automatic dot blot system with a 256-solid pin configuration (Kakengeneqs Inc., Chiba, Japan). Two sheets of membrane were prepared for one set of experiment. Spotted membranes were washed in TBS for 10 min, and blocked with 0.5% casein (Sigma) for 1 hr at RT. One membrane was then reacted with non-diluted KU-Lu-5 hybridoma supernatant, and the other membrane was reacted with antibody diluting solution [20-times diluted 0.5% casein with 0.1% Tween 20 added TBS (TBS-T)] for 30 min at RT. After rinsing in TBS-T 3 times for 5 min each, membranes were incubated with 1,000-times diluted horseradish peroxidase-conjugated rabbit anti-mouse IgG polyclonal antibody (Dako) for 30 min at RT. Finally, signals were developed with Immobilon Western reagent (Millipore Corp.). The data were analyzed using DotBlotChipSystem Ver. 4.0 (Dynacom Co., Ltd., Chiba, Japan). Each normalized signal was presented as the ratio of the positive intensity versus the negative background intensity.

### 8. Statistical analysis

Serum CAXII levels in patients with lung cancer and healthy controls were statistically analyzed using the Mann-Whitney *U*-test. Sensitivity, specificity, and predictive values were calculated with the SPBS software package (Ver. 9.42 for Windows) for each variable at a corresponding cut-off. Discriminant function analysis was performed to classify patients in the “lung cancer” vs. “healthy control” group, according to the status of the biomarkers, using the SPBS software package. The area under the curve (AUC) and best cut-off point were calculated employing receiver operating characteristic (ROC) analysis. Results were considered significant when *P*<0.05.

## Results

### 1. Confirmation of antibody titer in mouse sera

The antibody titer was tested by IHC with 1,000-times diluted sera of immunized mice as the first antibody on AMeX-fixed A549 cells. As a result, the sera from immunized mice contained antibodies that reacted with various components of A549 cells.

**Figure 1:**
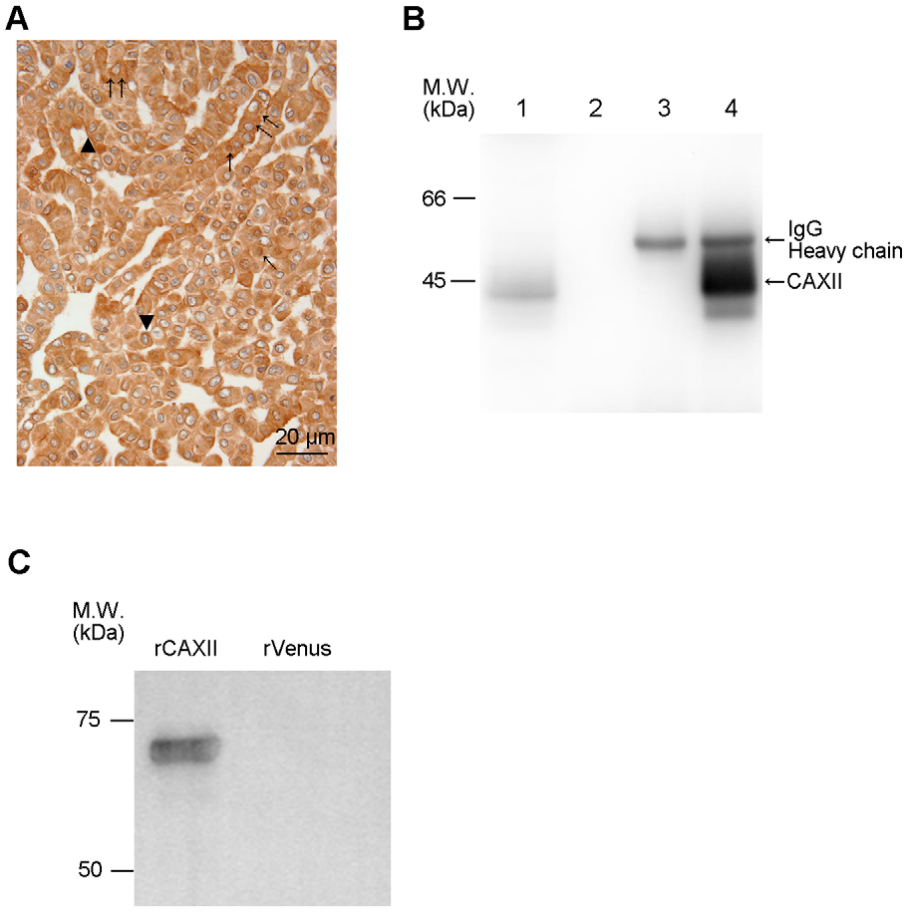
Production of anti-CAXII monoclonal antibody and its antigen identification. (A) The antibody titer was tested immunohistochemically using 1,000-times diluted sera of immunized mice as the first antibody on AMeX-fixed A549 cells, which were used as an immunogen. The sera of immunized mice contained antibodies that reacted with various cell components, such as the nucleus (↑), plasma membrane (▴), and cytoplasm (↑↑). (B) Immunoprecipitation with KU-Lu-5 antibody. Immunoblot analysis using KU-Lu-5 hybridoma supernatant as the first antibody [ lane 1: A549 lysate, lane 2: A549 lysate combined with protein G, lane 3: KU-Lu-5 antibody combined with protein G, lane 4: A549 lysate combined with KU-Lu-5 antibody]. Lanes 2 to 3 are negative controls, and immunoprecipitated product with KU-Lu-5 antibody was detected in lane 4 (↑). (C) Confirmation of identified antigen protein. KU-Lu-5 antibody reacted with recombinant CAXII protein (64 kDa), but not with recombinant Venus protein.

Using AMeX-fixed A549 cell preparations for the immunohistochemical screening of hybridomas, we finally established 188 MoAbs in total and a further study was performed with the KU-Lu-5 clone, which showed intense staining in A549 cells (Fig. 1 A).

### 2. Identification of antigen protein

In order to identify the antigen protein recognized by the KU-Lu-5 antibody, we performed IP with lysate from A549 cells. The results of IP are shown in Fig. 1 B, C. The antigenic protein was observed at roughly 40 kDa. To determine the antigenic protein recognized by KU-Lu-5 antibody, we excised and collected the spot from the Zn-stained gel, and proceeded with in-gel digestion. After analysis employing a MALDI-TOF/TOF MS and a MASCOT search, the protein was determined as isoform 2 of carbonic anhydrase XII (CAXII, accession: IPI00221392), which is composed of 343 amino acids with a predicted M.W. of 38,384 Da. The result was confirmed by immunoblot analysis with recombinant CAXII protein using KU-Lu-5 hybridoma supernatant as the first antibody (Fig. 1 D). The immunoglobulin isotype of KU-Lu-5 antibody was determined as IgG_1_, κ.

### 3. Immunoblot analysis

**Figure 2:**
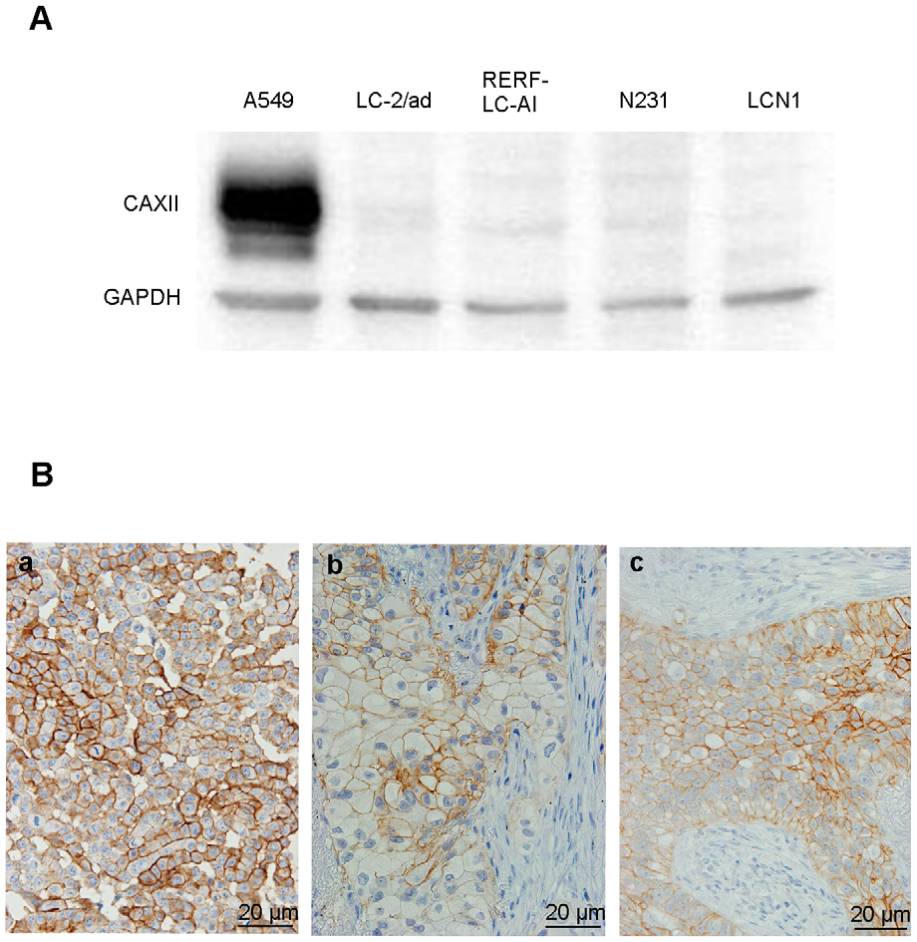
Expression of CAXII antibody in lung cancer cell lines and tissues. (A) Immunoblot analysis of CAXII in lung cancer cell lines. CAXII was detected as an approximately 40-kDa protein with A549 cells. (B) Immunostaining of CAXII in A549 cells (a), adenocarcinoma (b), and squamous cell carcinoma (c) of the lung, and each showed membranous staining of CAXII.

Expression of CAXII was detected only in A549 cells as a roughly 40-kDa protein, and no clear band was detected in other cells used in this study (Fig. 2 A).

### 4. Immunohistochemical staining for CAXII

Immunohistochemically, membranous expression of CAXII was observed only in A549 cells (Fig. 2 B). Membranous staining was detected in 2 of the 28 ADs (7.1%) and in 2 of the 9 SCCs (22.2%) (Fig. 2 C, D).

### 5. Serum CAXII in patients with lung cancer

**Figure 3:**
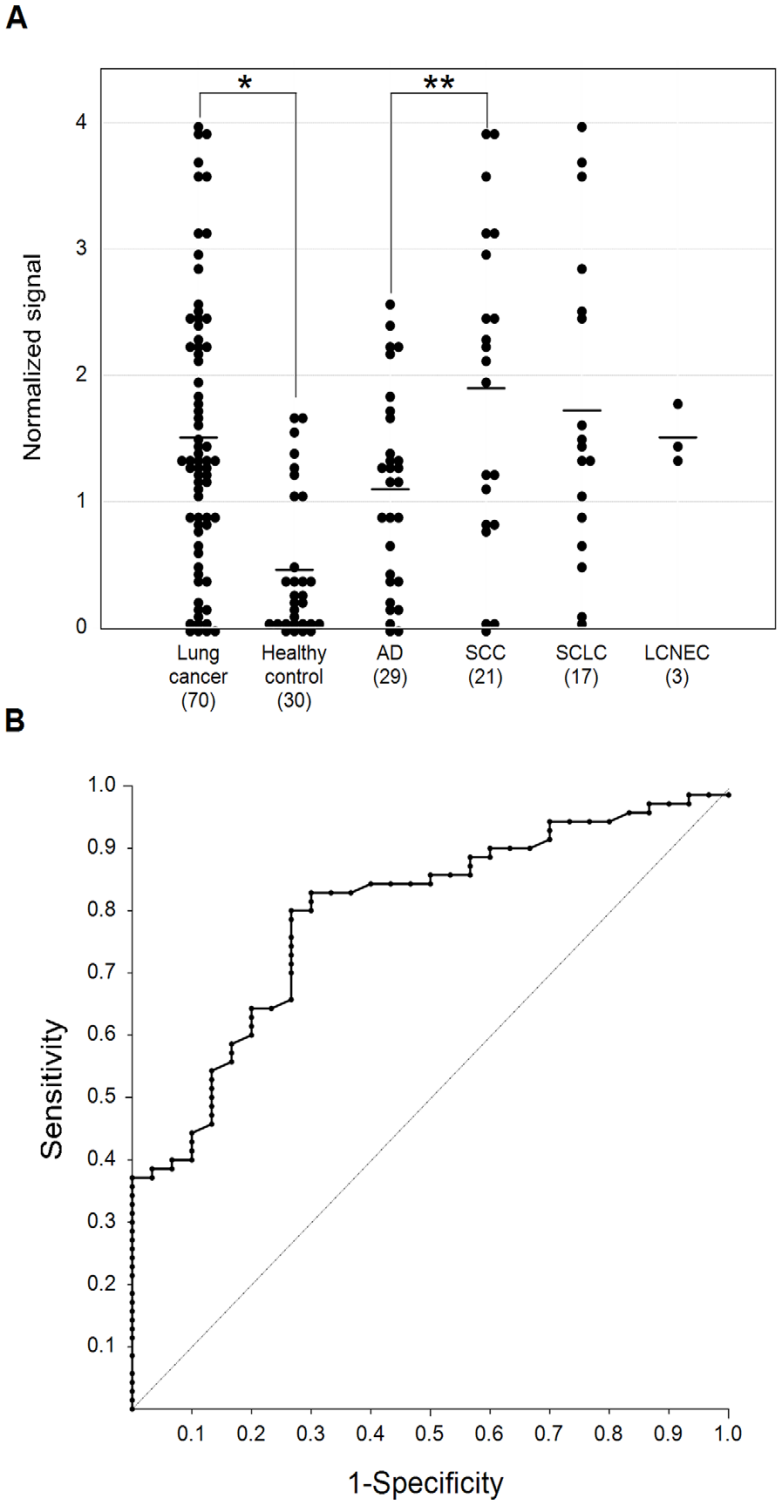
Serum CAXII levels in patients with lung cancer and healthy controls in the training set. Serum CAXII levels in patients with lung cancer and healthy controls. (A) The median CAXII level in the sera from healthy controls was 0.29, and that in sera from lung cancer patients was 1.52. Serum CAXII levels were significantly higher in lung cancer patients (*P<0.001). Furthermore, serum CAXII levels were higher in SCCs than ADs (**P = 0.0381). (B) Receiver-operating characteristic curve analysis of CAXII as a serum marker for lung cancer. The corresponding areas under the curves were 0.794 for CAXII. With a 70.0% specificity, the sensitivity of CAXII for lung cancer was 82.9%, at a cut-off value corresponding to 0.387.

**Figure 4:**
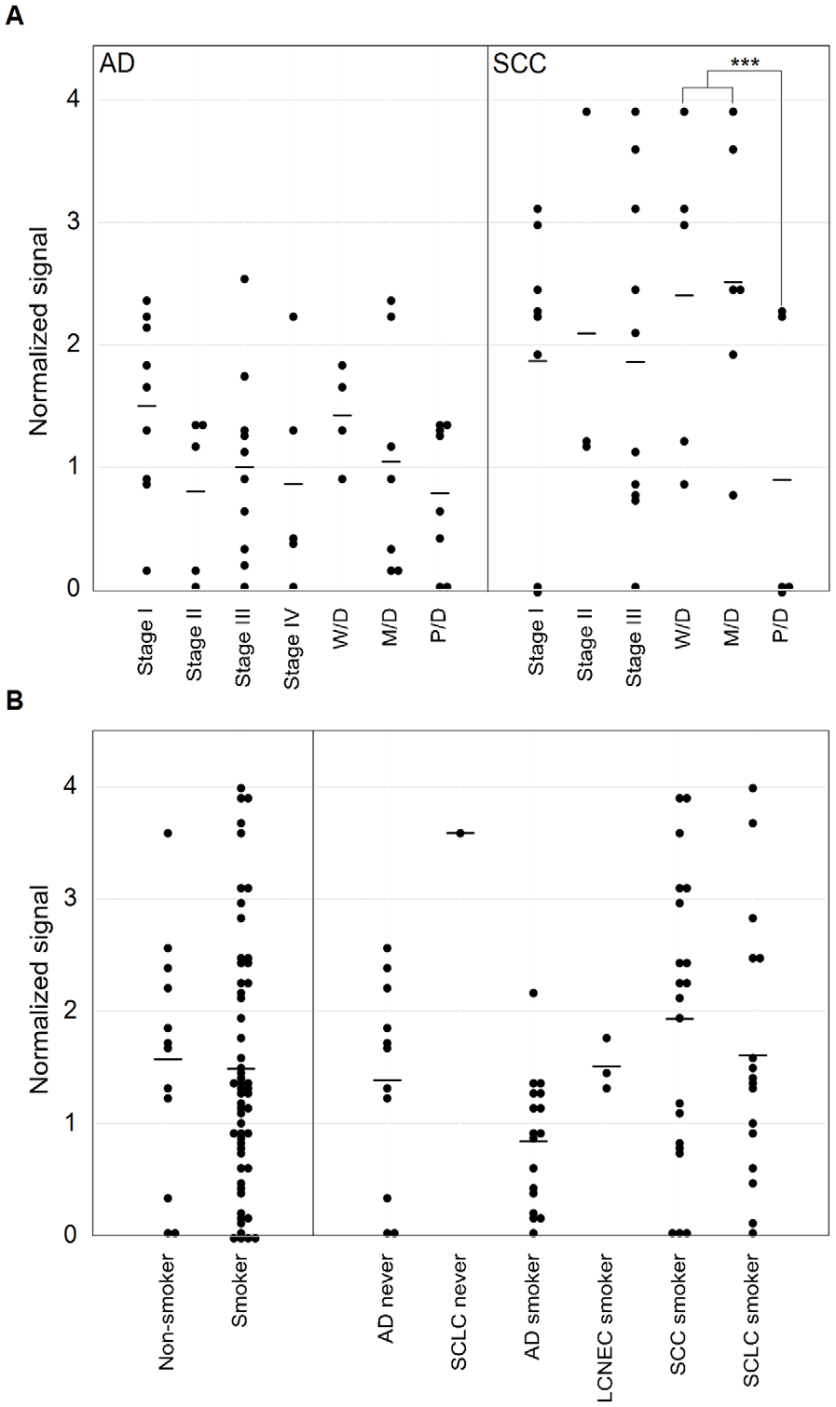
Correlation between serum CAXII levels and patients' clinicopathological characteristics. (A) CAXII levels in sera from patients with ADs and SCCs with a focus on the stage and differentiation. In SCCs, CAXII levels were significantly higher in well- and moderately differentiated tumors than in poorly differentiated ones (***P = 0.0272). In ADs, no significant difference based on the differentiation extent was detected. (B) Smoking history in lung cancer patients. The median CAXII level in the sera from non-smokers was 1.56, and that in smokers was 1.54, showing no significant difference.

**Figure 5:**
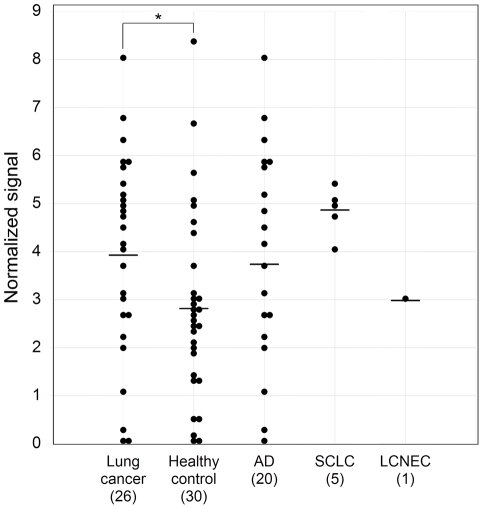
Serum CAXII levels in patients with lung cancer and healthy controls in the validation set. To confirm the utility of serum CAXII levels as a sero-diagnostic marker, 56 additional sera were analyzed by dot blot analysis as a validation study. The serum CAXII levels were also significantly higher in lung cancer patients than in healthy controls (P = 0.030). Relative values of serum CAXII levels ranged from 0.000 to 8.023 (median: 3.921) in lung cancer patients, but 0.000 to 8.331 (median: 2.806) in healthy controls.

The serum CAXII levels were significantly higher in lung cancer patients than in healthy controls in the training set (P<0.0001). Relative values of serum CAXII levels ranged from 0.101 to 4.01 (median: 1.520) in lung cancer patients, but 0.006 to 1.679 (median: 0.290) in healthy controls (Fig. 3 A). In lung cancer, CAXII serum levels of SCC patients were significantly higher than those of AD patients (P = 0.03) (Fig. 3 A). The area under the ROC curve (AUC) between lung cancers and healthy controls was 0.794 (Fig. 3 B). When an optimal cut-off value of 0.387 for CAXII was applied, the diagnostic sensitivity and specificity for lung cancer were 82.9 and 70.0, respectively, and the negative and positive predictive values were 0.617 and 0.863, respectively. Furthermore, within SCCs, serum CAXII levels were significantly higher in patients with well- and moderately differentiated tumors than those with poorly differentiated ones (P = 0.027) (Fig. 4 A), and tended to be higher in patients with a tumor size of less than 3 cm rather than more than 3 cm (P = 0.0538). However, there was no difference in the smoking history of patients (Fig. 4 B). CAXII levels in stage I, II, and III ADs were 1.501, 0.704, and 1.001, respectively, and CAXII levels in stage I, II, and III SCCs were 1.764, 2.093, and 1.854, respectively. These data were summarized in Table 2. No relations between the CAXII serum levels and tumor stage or presence of metastasis were identified for either ADs or SCCs. To further confirm the utility of serum CAXII levels as a sero-diagnostic marker, 56 additional samples of sera were analyzed by dot blot analysis as a validation study. The serum CAXII levels were also significantly higher in lung cancer patients than in healthy controls in the validation set (P = 0.030). Relative values of serum CAXII levels ranged from 0.000 to 8.023 (median: 3.921) in lung cancer patients, but 0.000 to 8.331 (median: 2.806) in healthy controls (Fig. 5). When an optimal cut-off value of 3.086 for applied, the diagnostic sensitivity and specificity for lung cancer were 65.4 and 70.0, respectively.

**Table 2:** Serum CAXII levels in ADs and SCCs.

		Average value (AD)	Average value (SCC)
Stage	I	1.501	1.764
	II	0.704	2.093
	III	1.001	1.854
	IV	0.654	0.000
Tumor differentiation	Well	1.424	2.403
	Moderate	1.046	2.511
	Poor	0.727	0.742

## Discussion

In this study, aiming to discover useful sero-diagnostic markers for lung cancer, we generated monoclonal antibodies using lung AD-derived A549 cells as antigens. From the obtained 188 antibodies, we focused on an antibody recognizing CAXII, and explored its clinical utility as a sero-diagnostic marker for lung cancer. This random immunization method is expected to yield antibodies against tumor-specific proteins with post-translational modifications, which are difficult to obtain by conventional immunization methods. Actually, several authors have reported that monoclonal antibodies generated by this method are useful as diagnostic and prognostic markers for cancers [5], [17], [19]. Battke *et al.* [20] established a 6A10 antibody recognizing CAXII using a similar immunization methodology. However, the obtained antibodies were limited to those only reacting with cell surface antigens because of using flow cytometry for the screening of hybridomas. In the present study, the hybridomas were immunohistochemically screened which facilitated the obtaining of antibodies reacting with tumor-associated proteins localized in several intra-cellular compartments. The CAs constitute a family of ubiquitous enzymes with important roles in many physiological and pathological processes which reversely catalyse the conversion of CO_2_+H_2_O to HCO_3_^−^ and H^+^, contributing to regulation of the intracellular pH [6]–[11], [19]. Several clinical studies have shown a clear relationship between high CAXII expression levels in tumor cells and a favorable prognosis.

Watson *et al.* [12] reported that CAXII was expressed in 75% of invasive breast carcinoma cases, and was significantly associated with a lower histological grade (P = 0.001), positive estrogen receptor status (P<0.01), and negative epidermal growth factor receptor overexpression (P<0.001). Using univariate analysis, CAXII-positive tumors were associated with a lower relapse rate (P = 0.04) and a better OS (P = 0.01). On the other hand, although 98% of astrocytomas were immunohistochemically positive for CAXII, higher CAXII expression levels were correlated with a higher histological grade and a poorer patient outcome either by univariate (P = 0.010) or multivariate (P = 0.039) survival analysis [9].

An immunohistochemical study of the expression of CAXII in lung cancer was reported by Ilie *et al.* [13]. CAXII overexpression was observed in 105/555 cases, and was significantly associated with a better differentiation (P = 0.015) and SCC histological type (P<0.001). Furthermore, high CAXII expression was also significantly correlated with better overall and disease-specific survival. From our results, CAXII levels were higher in sera from SCC patients than ADs (Fig. 3 A). Also, they correlated more favorably with differentiation (Fig. 4 A). Integrating these results, CAXII may not only be a candidate tissue marker, but also a sero-diagnostic marker for lung cancer.

Although the association of CAXII expression and clinicopathologic factors and patient outcome in different tumors has been reported, to our knowledge, no study concerning the serum CAXII protein levels or its autoantibody levels in patients with tumors has been reported.

To confirm the possibility of CAXII as a sero-diagnostic marker, we measured its serum levels in patients with lung cancer and healthy controls. We demonstrated that the CAXII protein flowed out into the sera and its levels in patients with lung cancer were significantly higher than in healthy controls in both the training set (P<0.0001) and validation set (P = 0.030). It is possible that the gap in the P-value between the training set and validation set is caused by the fact that serum levels of CAXII of SCC patients were generally higher than those of patients with other histologies, and the validation set included no SCC case. Taken together, the serum CAXII levels should be applicable markers discriminating lung cancer patients from healthy controls. Currently, CT scan or chest X-ray is the main method of lung cancer screening [21]. Mazzone et al. suggested that blood and breath tests should also be included for lung cancer screening, because they are both easy to perform and free of risks related to test administration [21], [22]. In this study, we analyzed CAXII levels in sera from lung cancer patients and healthy controls using monoclonal antibody, and our results suggested that the serum CAXII level was a useful sero-diagnostic marker for lung cancer.
